# Synchronisation of Neural Oscillations and Cross-modal Influences

**DOI:** 10.1016/j.tics.2020.03.003

**Published:** 2020-06

**Authors:** Anna-Katharina R. Bauer, Stefan Debener, Anna C. Nobre

**Affiliations:** 1 Department of Experimental Psychology, Brain and Cognition Lab, Oxford Centre for Human Brain Activity, Department of Psychiatry, Wellcome Centre for Integrative Neuroimaging, University of Oxford, UK; 2 Department of Psychology, Neuropsychology Lab, Cluster of Excellence Hearing4All, University of Oldenburg, Germany

**Keywords:** multisensory, cross-modal influence, neural oscillations, phase reset, neural entrainment, causal inference

## Abstract

At any given moment, we receive multiple signals from our different senses. Prior research has shown that signals in one sensory modality can influence neural activity and behavioural performance associated with another sensory modality. Recent human and nonhuman primate studies suggest that such cross-modal influences in sensory cortices are mediated by the synchronisation of ongoing neural oscillations. In this review, we consider two mechanisms proposed to facilitate cross-modal influences on sensory processing, namely cross-modal phase resetting and neural entrainment. We consider how top-down processes may further influence cross-modal processing in a flexible manner, and we highlight fruitful directions for further research.

## Highlights

Electrophysiological studies suggest that cross-modal influences in sensory cortices are mediated by the synchronisation of neural oscillations through phase-resetting and neural entrainment mechanisms.
Low-frequency neural oscillations in the delta, theta, and lower alpha ranges are suggested to provide temporal windows for cross-modal influences.
Top-down factors, such as task goals and expectations, may modulate multisensory processing as well as neural oscillations.
Bayesian computational modelling provides a new approach to probe cross-modal influences.

## Cross-modal Activations in Unimodal Cortices

In our daily life, we continuously receive information from different sensory modalities, such as sight, sound, and touch. Think of a glass falling and breaking on the floor or footsteps of a person walking into a room. Incoming sensory signals are often interrelated and provide complementary evidence about our environment. To form a rich and adaptive understanding of our environment, signals from different modalities can influence one another. When originating from common sources, spatial proximity and temporal correlation may lead to integration into multisensory representations. To shed light on the mechanisms of **cross-modal influences** (see Glossary) and integration, in this review we consider whether and how oscillatory activity in cortical areas may contribute. Specifically, we use the term ‘cross-modal influences’ to express how the processing of sensory stimulation in one modality affects the neural processing or behaviour associated with another sensory modality [1,2].

The first cortical regions that process incoming visual, auditory, and somatosensory information are the primary visual (V1), auditory (A1), and somatosensory (S1) cortices. According to the standard understanding of perceptual systems, processing of incoming sensory information evolves from the extraction of simple features in these highly specialised primary cortical structures through progressively more integrated representations in unimodal and multimodal associative regions [3]. Outputs from these loosely hierarchical sensory networks then converge in multisensory and higher order cortical regions, in particular the superior temporal sulcus (STS), intraparietal sulcus (IPS), and prefrontal cortical regions (PFC) [4,5]. Traditionally, it has been believed that the merging of sensory information from different modalities in cortex occurred exclusively in these multisensory and higher order regions.

However, several human and animal studies have provided convincing evidence that cross-modal cortical influences can occur much earlier, even at the level of the primary sensory cortices [2,4,6., 7., 8., 9.]. These early cross-modal influences in primary sensory cortices are modulatory in nature. Rather than driving neuronal activity, sensory signals from another modality change the cortical excitability to the signals in the dominant modality [7,10]. These findings have prompted a revision of our understanding of unimodal cortical regions and of the pathways that enable cross-modal influences and the integration of sensory information in cortex. In addition to indirect cross-modal influences through higher order multimodal cortical regions (STS, IPS, and PFC) [4,5,7], there are pathways through multimodal subcortical regions (e.g., superior colliculus and the pulvinar nucleus of the thalamus) [10., 11., 12.], and possibly direct lateral connections between unimodal cortices [13]. In principle, multiple pathways may coexist, and involvement of different pathways may depend on the specific stimulus parameters, task demands, and presence of top-down factors.

## Neural Oscillations as a Substrate of Cross-modal Influences

Recently, several studies promoted the notion that the synchronisation of **neural oscillations** may be an important mechanism for enabling cross-modal influences by facilitating the transfer of information across sensory modalities [5,8] (for a recent review, see [2]). Neural oscillations reflect the rhythmic fluctuations of excitability in neuronal ensembles related to the dynamics of the circuits in which ensembles are embedded as well as the kinetics of their ionic channels [14]. Rhythmic transitions between states of relatively high and low excitability can be characterised in terms of their frequency, amplitude, and **phase** [14]. The phase indicates the particular point along the oscillatory cycle between 0 and 2 pi, corresponding to the peak, trough, or somewhere in between. Sensory inputs coinciding with the high-excitability state elicit stronger neural responses, whereas inputs coinciding with the low-excitability phase are attenuated (e.g., [15]). This suggests that there are phases at which the processing of sensory information is optimised. Indeed, several studies have shown that behavioural performance across various tasks and in different sensory modalities fluctuates according to the phase of ongoing neural oscillations (for a review, see [16]). While amplitude and frequency can also impact neural excitability and behavioural performance [17], this review mainly focuses on phase-dependent effects.

**Figure 1:**
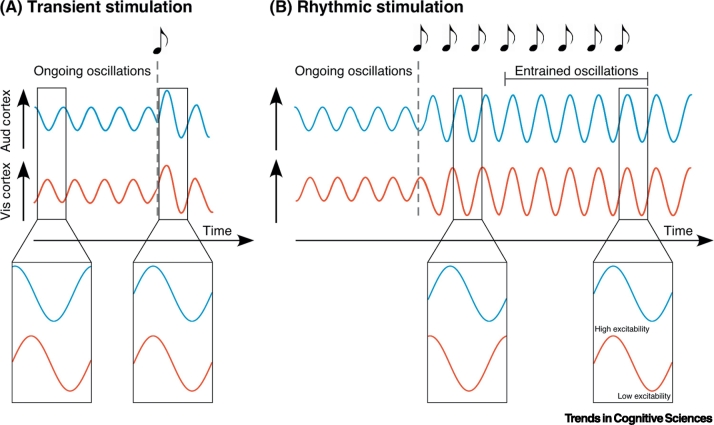
Basic Principles of Phase Resetting (A) and Neural Entrainment (B) Mechanisms. (A) Phase reset results from a single transient event (e.g., sound or flash of light) that ‘resets’ the phase of ongoing neural oscillations. Schematic representation of phase realignment of neural oscillations in the auditory cortex (blue) and visual cortex (red) due to a transient event. (B) Phase entrainment occurs as the result of a rhythmic stimulus gradually shifting the phase of the neural oscillation. Schematic representation of phase realignment of ongoing neural oscillations in the auditory cortex (blue) and visual cortex (red) due to external rhythmic stimulation. For both transient and rhythmic stimulation, the phase of ongoing neural oscillations aligns to the driving stimulus, thereby modulating the excitation-inhibition cycle of the neural oscillation.

Neural oscillations have been repeatedly suggested to facilitate cross-modal influences between primary visual, auditory, and/or somatosensory areas (e.g., [10,18]). In general terms, two brain regions are considered to be synchronised or ‘phase coherent’ when there is a constant phase relationship between the two modality-specific activations over time [19,20]. Previous theoretical and empirical work suggests that the synchronisation of ongoing neural oscillations is essential for determining the selection and routing of information both within and between cortical areas [2,5,8,19,20]. Whereas signals occurring in synchrony with high-excitability phases are effectively exchanged, asynchronous signals or signals linked to low-excitability phases are likely impeded. Synchronisation of oscillatory activity is usually considered to come about through one of two different mechanisms: **cross-modal phase resetting** ([10]; for a review, see [21]) or **neural entrainment** [15] (for a schematic representation, see Figure 1).

### Cross-modal Phase Reset

The concept of phase resetting was first introduced to intramodal processing [22] and has sparked interest in the non-invasive study of event-related brain dynamics [23] (Box 1). Cross-modal phase reset refers to the process by which the phase of ongoing neural oscillations in one sensory modality can be ‘reset’ by a transient event in another sensory modality (Figure 1A). The benefits of synchronising neural processing by transient events may bring similar benefits to cross-modal influences. In this case, a single salient or attended external (or internal) stimulus can ‘set’ the phase of a neural oscillation to a particular state of excitability within another sensory modality [24].

Box 1 **Phase Resetting Mechanism**

The phase of an ongoing neural oscillation can be reset by a salient external (or internal) event. In visual tasks, salient visual events result in fluctuations of behavioural accuracy and reaction times in the theta and alpha frequency bands [37,106., 107., 108., 109.]. In audition a recent study further demonstrated behavioural fluctuations of auditory target detection performance in the theta frequency range in response to a salient auditory tone [110]. Phase resets have also been observed across sensory modalities at different frequency bands in response to salient visual cues (e.g., red disk) [41], brief auditory tones (e.g., white noise burst [28]), or in response to somatosensory stimulation [10] (see also Table 1 in the main text). Internal events can also reset the phase of periodic behavioural performance. Recent studies have shown that internally generated motor events [111,112] can reset performance fluctuations on visual tasks (for review, see [113]). Overall, the emergence of behavioural periodicities time-locked to a reset event is a prime indicator of the involvement of neural oscillations [21,27,38].

On a physiological level, difficulties may arise in determining a genuine phase reset. A pure phase reset is characterised by a stimulus-induced phase realignment of oscillatory activity without any concomitant change in power [23]. In the brain, phase resets by transient events are unlikely to be pure. Stimuli evoke a time-locked neural response characterised by an increase in power across a range of frequencies, thus also leading to an increase in phase concentration measures [23,114]. Cross-modal influences by phase reset involve the evoked response to a salient unimodal stimulus in one modality resetting the phase of oscillatory activity in another modality. Separating evoked responses from phase resets can be problematic. The high spatial and temporal resolutions of intracranial recordings make it easier to distinguish evoked responses from phase reset (e.g., [32,41]) than is possible using standard scalp EEG data [21]. To overcome limitations in non-invasive studies, it is necessary both to increase the spatial resolution of the recordings by using dense sampling and computing the sources of the cortical oscillations (e.g., [28,40]) and to increase the temporal resolution of analysis methods to investigate the instantaneous phase of oscillatory activity before, during, and after a transient stimulus.

Alt-text: Box 1

Cross-modal phase reset was first described in nonhuman primate studies investigating auditory cortical responses to auditory and nonauditory stimuli [10,25]. For instance, somatosensory stimulation of the median nerve preceding a brief auditory tone changed the phase of ongoing oscillations in A1. Moreover, the response to the subsequent auditory tone was modulated by the reset phase of auditory neural oscillations [10]. Somatosensory modulation of auditory oscillations led to the auditory input arriving during a high-excitability phase, resulting in an enhanced auditory cortical response. The authors suggested that the functional role of this cross-modal phase reset is to aid or impede the selection of paired cross-modal signals according to task demands, by either aligning or misaligning the high-excitability states across sensory modalities [10]. Subsequent studies in nonhuman primates further showed that oscillatory phase in the auditory cortex can be reset by visual input, and vice versa [24., 25., 26.].

In humans, most studies investigating cross-modal phase reset focus on the interaction between the auditory and visual modalities using a range of different experimental tasks and imaging techniques (Table 1). Evidence for cross-modal auditory-to-visual influences in the primary visual cortex is provided by studies demonstrating that auditory input can reset activity in visual cortex and modulate visual perceptual performance [27., 28., 29., 30., 31., 32., 33., 34., 35., 36.]. For instance, a behavioural study demonstrated that the presentation of a short auditory tone resulted in the rhythmic modulation of visual target detection performance persisting for up to 6 s after tone onset [27]. The study was able to probe the periodicity in behavioural performance by prompting a perceptual decision at different time points relative to the phase-resetting event (see also [37]). The distance between these time points over the various trials yields a sampling rate. The sampling rate constrains how well it is possible to resolve the frequency of the behavioural oscillation, and insufficient sampling can be a major limitation in some studies. For example, by sampling behaviour every 500 ms, only oscillations ~1 Hz can be resolved. Periodicities in purely behavioural measures are a strong indicator for the involvement of neural oscillations (e.g., [16,38]), but they remain indirect.

**Table 1:** Empirical Reports of Cross-modal Phase Reset^a^

First author	Refs	Year	CM influence	Method	Species	Reset event	Affected oscillations	Perceptual consequence
Fiebelkorn	[27]	2011	A to V	Beh	Human	Short tone	Low frequency	Periodic modulations of target detection rate
Naue	[28]	2011	A to V	EEG	Human	White noise burst	Beta	None reported
Diederich	[29]	2012	A to V	Beh	Human	White noise burst	Beta, gamma	Periodic modulations of saccadic response times
Romei	[30]	2012	A to V	EEG-TMS	Human	Short tone	Alpha	Periodic modulation of TMS-induced phosphene perception
Fiebelkorn	[31]	2013	A to V	EEG	Human	Short tone	Delta to beta	Periodic modulations of target detection rate
Mercier	[32]	2013	A to V	ECoG	Human	Short tone	Theta to gamma	None reported
Diederich	[33]	2014	A to V	EEG	Human	Short tone	Theta, alpha	Periodic modulations of saccadic response times
Cecere	[34]	2015	A to V	EEG-tACS	Human	Short tone (sound-induced double-flash illusion)	Alpha	None reported
Keil	[35]	2017	A to V	EEG	Human	Short tone (sound-induced double-flash illusion)	Alpha	None reported
Plass	[36]	2019	A to V	ECoG	Human	Short tone	Theta, alpha, beta	None reported
Senkowski	[39]	2005	V to A	EEG	Human	AV grating/short tone	Gamma	Faster behavioural responses
Kayser	[25]	2008	V to A	LFP	Macaque	Naturalistic scenes	Alpha	None reported
Thorne	[40]	2011	V to A	EEG	Human	AV dash/tone streams	Theta, alpha	Faster behavioural responses
Mercier	[41]	2015	V to A	ECoG	Human	Red disk	Delta, theta	None reported
Perrodin	[26]	2015	V to A	LFP	Macaque	Naturalistic scenes	Theta	None reported
ten Oever	[42]	2015	V to A	EEG	Human	Circle	Delta, alpha	None reported
Lakatos	[10]	2007	T to A	CSD	Macaque	Median nerve stimulation	Delta, theta, gamma	None reported
Lakatos	[24]	2009	A to V V to A	CSD	Macaque	Short tones, flicker	Theta, gamma	None reported

a Abbreviations: A, auditory; AV, audiovisual; Beh, behavioural; CSD, current source density; ECoG, electrocorticography; EEG, electroencephalography; LFP, local field potential; MEG, magnetoencephalography; T, tactile; tACS, transcranial alternating current stimulation; TMS, transcranial magnetic stimulation; V, visual.

Direct evidence for auditory-induced visual phase reset comes from a study using electrocorticography (ECoG; [32]). Patients undergoing epilepsy surgery performed a simple detection task, in which they responded to unimodal auditory or visual stimuli or to bi-modal auditory-visual stimuli. Analysis of activity in visual cortex after unimodal auditory stimulation revealed an increase in the phase alignment of visual oscillations in the theta (4–8 Hz) and alpha band (8–12 Hz), as measured using intertrial phase coherence (ITPC). A similar pattern of results was obtained using non-invasive recordings in healthy volunteers. Unimodal auditory stimuli led to phase resetting of visual alpha activity [30]. In addition to the physiological effects, behavioural periodicities were also observed. Phosphene perception induced by transcranial magnetic stimulation (TMS) of the visual cortex also fluctuated around 10 Hz, time locked to sound onset.

In many natural situations, visual events precede, or even generate, sound events (e.g., lip movements typically precede vocal sounds), which has motivated studies investigating cross-modal visual-to-auditory influences [39., 40., 41.]. In an ECoG study participants were presented with unimodal or bi-modal visual and auditory stimuli while electrocorticography was recorded from the auditory cortex [41]. Visual stimuli reset oscillatory activity in auditory cortex in the delta (1–4 Hz) and theta bands. Converging evidence for visual-to-auditory influences comes from scalp electroencephalography (EEG) recordings. In an auditory frequency-discrimination task, participants were presented with repeated pairings of asynchronous visual and auditory stimuli and judged the pitch of the final tone relative to the preceding ones [40]. The lag between visual and auditory stimuli varied across trials. The recordings showed visually induced phase resetting of auditory signals in the theta and alpha frequency ranges. Effects were strongest when visual stimulation preceded the auditory stimulation by 30 to 75 ms. Increases in ITPC were further associated with faster reaction times.

Table 1 summarises the results from human and nonhuman primate studies on cross-modal phase reset, providing evidence for phase resetting based on visual, auditory, and somatosensory stimuli. The studies report periodic fluctuations in behaviour as well as in neural activity following a cross-modal phase reset. Furthermore, these periodic fluctuations have been observed across a range of frequencies, indicating that cross-modal phase reset may operate across multiple timescales.

### Cross-modal Entrainment

Neural activity and behavioural performance are also sensitive to (quasi-)rhythmic external stimulation. Many natural stimuli, such as speech and music, follow a regular rhythm that can entrain oscillatory brain activity (Box 2; for recent reviews on neural entrainment, see [43,44]). According to dynamical systems theory, entrainment is defined as the synchronisation of two (or more) self-sustained oscillators [45]. In cognitive neuroscience, neural entrainment is most commonly described as the gradual phase alignment of an ongoing neural oscillation to external rhythmic or quasi-rhythmic stimulation [15]. Oscillatory entrainment has been noted predominantly in the delta and theta frequency ranges [15]. Such entrainment of neural oscillations to rhythmic stimulation has been considered as a powerful neural mechanism to enhance the processing of predicted future events (for a review, see [46]). Neural entrainment has been proposed to support periodic perceptual modulations, where behavioural performance is generally better at on-beat relative to off-beat times (e.g., [47,48]). High-excitability phases of neural oscillations come to be aligned with the onset of the regular stimulation, thus conferring behavioural advantages (e.g., [15,38,49., 50., 51.]). In the auditory modality, neural entrainment effects have been observed in response to discrete and continuous sounds [38,49,52., 53., 54., 55., 56.], speech [57,58], musical rhythms [59], and even to perceptually subthreshold stimuli [60]. Furthermore, visual oscillations synchronised to the regular presentation of visual stimuli [50,51,61,62]. Similar to phase reset, neural entrainment has also been proposed to mediate influences between sensory cortices [24,43,63].

Box 2 **Entrainment Mechanism**

Psychophysical experiments were the first to demonstrate that behavioural performance ebbs and flows in pace with periodic stimulation [115]. Auditory perceptual identification and discrimination were enhanced when stimuli were presented in time with a sequence of stimuli separated by a constant interval ([116., 117., 118.]; but see [119]). Such observations led to the ‘**dynamic attending theory**’ (DAT), which proposed that isochronous rhythmic stimulation entrained an attention-related function of expectancy that resulted in better performance for stimuli temporally predicted by the previous stimulation [118,120]. Recordings in sensory cortices of nonhuman primates later showed synchronisation of oscillatory activity to (quasi-)rhythmic stimulus streams, thereby providing a plausible basis for DAT [15,121]. Although the current review focuses on cortical mechanisms, entrainment is not restricted to cortical regions (e.g., [122]).

Entrainment requires that two oscillators interact through direct synchronisation [45,123]. When it comes to the sensory entrainment of brain activity, one oscillator is usually the external rhythmic (or quasi-rhythmic) input stream and the other is the neuronal ensemble, which displays intrinsic rhythmic changes in excitability. Synchronisation can be measured by the increase in phase coherence in M/EEG recordings at the driving stimulation frequency. Once the external stimulation ceases, the neural system will return to its default, characteristic intrinsic oscillatory frequency.

Establishing neural entrainment effects during ongoing rhythmic stimulation is not trivial, because phase locking can also arise from non-oscillatory sources. For example, in the case where a series of individual transient events is presented separated by constant, isochronous intervals; it is expected that each stimulus would evoke time-locked neurophysiological potentials. When analysed with Fourier-based methods, these would lead to increased power and phase coherence at the driving stimulus frequency, similar to what would be observed if real entrainment were occurring [124]. The fact that stimuli elicit evoked potentials does not negate them also eliciting entrainment, but separating the two effects remains a challenge.

Interestingly, the steady-state evoked potential method (SSEP) uses rapid isochronous presentation of stimuli at different frequencies to tag and individuate their respective neural responses [125]. By combining this method of stimulus presentation with frequency-based analyses, the method has generated rich insights into perceptual and attentional processing [125]. In light of the entrainment literature, it is interesting to consider whether steady-state sensory stimulation does not itself change the very nature of the neural processing it is intended to measure [126].

Neural entrainment should also be distinguished from resonance responses [127]. In contrast to neural entrainment, resonance describes the response of a system that does not exhibit self-sustained oscillatory activity, but that resonates briefly when stimulated [127]. Even a singular event can trigger a frequency-specific resonance response, which is reflected by an increased amplitude in the M/EEG [128]. This similarity in neural responses as measured by M/EEG makes it hard to differentiate pure oscillatory entrainment from resonance responses. While few studies fail to test explicitly for the different possible explanations, recent papers investigating responses to visual as well as auditory rhythmic stimulation argue for the involvement of oscillatory rather than evoked responses [62,129].

Given that different underlying mechanisms can result in similar phase and amplitude modulations during rhythmic stimulation as revealed by standard analysis methods, it is important to consider the prestimulus phase and/or the period after termination of sensory stimulation [123]. Indirect support for the existence of entrainment mechanisms comes from a recent study showing periodic fluctuations of behavioural performance that outlasted the rhythmic stimulation by several cycles [130]. In the brain, recordings from auditory cortex similarly showed persistence of oscillations in step with rhythmic stimulation sustained even after the stimulation ended [131].

Alt-text: Box 2

An increasing number of studies have investigated the role of neural entrainment in cross-modal processing [64., 65., 66., 67., 68., 69., 70., 71.]. For instance, a series of behavioural experiments tested the influence of auditory rhythmic stimulation on visually guided behaviour [66]. The regular presentation of short auditory tones (presentation rate: 1.67 Hz) influenced the temporal allocation of visual saccades [66]. In particular, saccade latencies were shorter when the target onset occurred on beat with the preceding auditory rhythm, relative to when the target onset occurred off beat.

In another study using scalp EEG recordings, visual targets were presented either on or off beat with a preceding slow auditory rhythm (1.3 Hz) [67]. Analysis of visual oscillatory activity at the time of visual target onset revealed phase differences in the lower beta band (13–20 Hz) depending on whether the target occurred in versus out of pace with the preceding auditory rhythm. Moreover, these phase differences were directly linked to target-related visual potentials. N100 amplitudes were larger over occipital sensors when visual targets were presented in time with the preceding auditory stream.

The classic example for a (quasi-)rhythmic visual-to-auditory influence is speech, because seeing the lip movements typically precedes hearing the corresponding voice. A recent EEG study compellingly demonstrated how lip movements entrain low-frequency neural oscillations in the delta and theta frequency ranges in visual and auditory cortices [70]. Participants observed audiovisual videos containing either matching or non-matching auditory and visual content in natural speech. Comparing the synchronisation of neural oscillations in visual and auditory cortices between conditions revealed enhanced neural entrainment effects for the matching speech condition, as measured by increased coherence. It would be interesting to investigate the extent to which such effects are purely stimulus driven or are also modulated by higher level factors, such as speech comprehension or task engagement (for a recent review, see [44]).

Both auditory and visual (quasi-)rhythmic stimulation can influence behavioural performance and neural processing across sensory cortices (see Table 2 for a summary of studies on cross-modal entrainment). Most studies so far have used regular auditory rhythms as the entraining sequence. This is not surprising given the abundance of auditory environmental stimuli that are intrinsically rhythmic in nature. Sounds naturally evolve over time and often contain a predictable temporal structure, which can provide a pacing signal for neural oscillations across multiple frequency bands [15,72]. So far, presentation rates used for entraining sequences have been exclusively in the delta and theta frequency ranges, which correspond to the time scales in which typical environmental rhythms, such as speech or biological motion, operate. An outstanding question is whether visual rhythms (other than speech stimuli) prove equally effective at entraining neural oscillations in the auditory cortex and at enhancing behavioural performance.

**Table 2:** Empirical Reports of Cross-modal Entrainment^a^

First author	Refs	Year	CM influence	Method	Entraining sequence	Affected oscillations	Perceptual consequence
Bolger	[64]	2013	A to V	Beh	Isochronous tone sequence (2 Hz) and classical music excerpts	–	Faster behavioural responses for salient metric positions
Brochard	[65]	2013	A to V	Beh	Syncopated rhythm (1.25 Hz)	–	Facilitated word recognition for on-beat times
Miller	[66]	2013	A to V	Beh	Isochronous tone sequence (1.67 Hz)	–	Faster saccadic responses for on-beat times
Escoffier	[67]	2015	A to V	EEG	Isochronous tone sequence (1.3 Hz)	Beta	None reported
Simon	[68]	2017	A to V	EEG	Amplitude modulated white noise (3 Hz)	Delta, theta, alpha	Periodic modulation of target detection rate
Barnhart	[69]	2018	A to V	Beh	Isochronous tone sequence (0.67 Hz and 1.5 Hz)	–	Faster behavioural responses for on-beat times
Park	[70]	2016	V to A	MEG	AV speech	Delta, theta	None reported
Megevand^b^	[71]	2019	A to V (V to A)	iEEG	AV speech	Delta, theta	None reported

a Abbreviations: V, visual; A, auditory; AV, audiovisual; Beh, behavioural; EEG, electroencephalography; iEEG, intracranial EEG; MEG, magnetoencephalography.

b Preprint.

### Distinct Roles of Cross-modal Phase Reset and Neural Entrainment

The two mechanisms, cross-modal phase reset and neural entrainment, have a similar functional outcome. They reorganise the phase of ongoing neural oscillations so that high-excitability phases across sensory modalities align to the timing of relevant events, resulting in enhanced neural and perceptual processing. However, these functional consequences come about through different means. While neural entrainment entails the gradual phase alignment of two (quasi-)rhythmic processes, phase reset involves a transient phase reorganisation due to a transient event. Hence, the two mechanisms may transmit distinct types of information across sensory modalities: either the timing of an expected stimulus (neural entrainment) or the timing of an external stimulus (phase-reset).

### Top-Down Control and Cross-modal Influences

In complementary literatures, we find substantial evidence that top-down attention-related processes, such as task goals or **temporal expectations**, modulate multisensory processing as well as neural oscillations ([42,73., 74., 75., 76., 77., 78., 79., 80.]; for a review, see [2]). It will be fruitful to explore whether and how **top-down processes** influence the synchronisation of signals across sensory modalities. In the case of phase resetting, the modulation of a neural response by a punctate stimulus would be likely to modulate its effect on an ongoing oscillatory signal. In the case of neural entrainment, one could imagine that changes in oscillatory power by selective attention [81] might modulate the ability of oscillatory signals to interact. Interestingly, when top-down information includes predictions about the temporal onset of events and enables temporal expectations, it may even be possible for internal signals to alter phase-reset or neural entrainment mechanisms directly.

A seminal nonhuman primate study showed how the task relevance of a specific modality can modulate the degree of neural entrainment between a (quasi-)rhythmic stimulus stream in that modality and oscillatory activity in primary sensory areas [15]. Macaque monkeys were exposed to concurrent visual and auditory near-rhythmic streams presented in anti-phase (each at 1.5 Hz). Attention to one of the two streams resulted in a relative increase in delta-phase synchronisation in the respective primary sensory cortex compared with when that same stream was unattended. This selectivity of modality-specific neural entrainment has also been observed in humans using ECoG and a related task design [82]. Participants were either presented with a regular or jittered stream of interleaved auditory and visual stimuli. When participants attended to one of the two streams, low-frequency delta oscillations became entrained to that modality, and the strength of entrainment was further dependent on the temporal predictability of the sensory stream. Although these studies target intermodal selection rather than cross-modal influences, they provide an excellent example of the proactive phase synchronisation of neural oscillations due to top-down factors in a cross-modal context.

One interesting EEG study suggested that temporal expectations related to the regular temporal co-presentation of visual and tactile stimuli led to increased phase coherence at the stimulation rate (delta) in somatosensory cortex [75]. In addition, when participants focused on either the visual or tactile stimuli, local power modulations were observed for visual alpha or somatosensory beta oscillations, respectively, in line with selective attention to that modality. Evidence for the modulation of cross-modal synchronisation was also obtained in an EEG study involving cued cross-modal temporal expectations [42]. In this study, a visual cue predicted the timing of an upcoming near-threshold tone. The visual cue was observed to reset low-frequency oscillations (around 1 Hz), resulting in their realignment to the onset of the auditory target. These two studies provide a promising base for future investigations of how top-down factors, and especially temporal expectations, may modulate or drive synchronisation of processing across the sensory modalities.

## Computational Principles of Cross-modal Influences

Recent advances in the field of computational modelling provide new insights into how the brain can correctly apportion incoming signals across various modalities to their events of origin, integrating and segregating across modalities accordingly. Recent psychophysical and neuroimaging studies support the theoretical proposal that the brain solves this problem through mechanisms approximating **Bayesian Causal Inference** [83., 84., 85., 86., 87., 88., 89.] (Box 3). Bayesian modelling has been successful in describing human perception in various cross-modal settings [88,89] and has been suggested as a framework to map neural processes onto distinct sensory computations in line with integration (or fusion), segregation, and causal inference [83].

Box 3 **Bayesian Causal Inference**

To make sense of the environment and incoming sensory signals, the brain must solve several computational problems. First, a brain needs to solve the causal inference problem [84,132]: do incoming sensory signals originate from a common source and, hence, provide complementary evidence about the environment or do they represent different sources? To solve this problem, the brain relies on several factors, such as temporal coincidence, spatial location, and structural congruency between incoming stimuli, and is further influenced by our prior knowledge and expectations [83]. Second, if a common cause is concluded, the brain must determine how information about the different sensory modalities should be integrated across the senses [133,134]. Behavioural and neuroimaging studies proposed that the human brain solves these computational problems optimally using a mechanism akin to Bayesian Causal Inference [83., 84., 85., 86., 87., 88., 89.]. Bayesian Causal Inference provides a rational strategy to arbitrate between information segregation versus integration in perception and cognition [83,84] (Figure IA–C). In the case of independent sources (C=2), incoming sensory signals from different modalities should be segregated (Figure IA). Under the assumption of a common cause (C=1), signals should be merged across sensory modalities (forced fusion, Figure IB). Critically, the brain cannot directly access the causal structure of our environment and has to infer whether incoming sensory signals originate from a common or two separate sources. To account for observers’ causal uncertainty, an estimate can be obtained by combining the forced-fusion and the unimodal segregation estimates under various causal structures using decisional strategies, such as model averaging, model selection, or probability matching [88] (Figure IC).

To probe whether Bayesian Causal Inference can account for human perception, previous studies mainly focused on spatial location tasks [85,88,89], but some recent studies have also used temporal designs [87]. In these studies, sensory stimuli are presented with varying degrees of either temporal or spatial disparity. Bayesian Causal Inference can explain perceptual judgements across the range of discrepancies, spanning a continuum, from fusion to partial integration to segregation (Figure ID–F) [88].

**Figure I:**
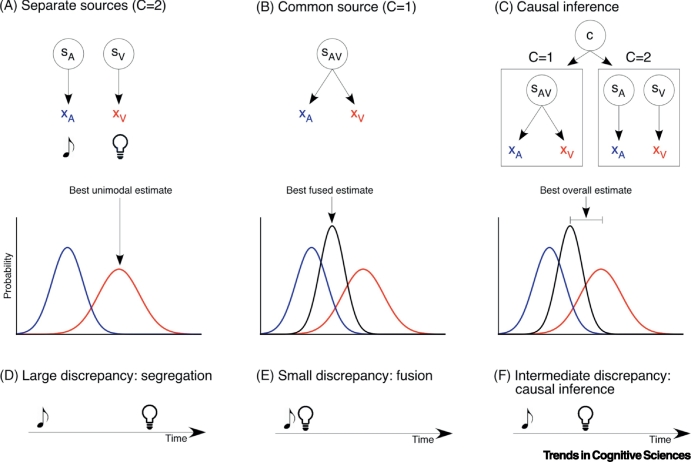
Computational Modelling of Cross-modal Interactions*.* (A–C) The first row depicts a schematic representation of different causal structures in the environment. S_A_, S_V_, and S_AV_ represent sources of auditory, visual, or cross-modal stimuli, and X_A_ and X_V_ indicate the respective sensory representations (e.g., time or location). The bottom row depicts the probability distributions of these sensory representations derived from the Bayesian model. (A) Assuming separate sources (C=2) leads to independent estimates for auditory and visual stimuli, with the optimal value matching the most likely unimodal response. (B) Assuming a common source (C=1) leads to fusion of the two sensory signals. The optimal Bayesian estimate is the combination of both auditory and visual input, each weighted by its relative reliability. (C) In Bayesian Causal Inference, the two different hypotheses about the causal structure (e.g., one or two sources) are combined, each weighted by its inferred probability given the auditory and visual input, known as model averaging. The optimal stimulus estimate is a mixture of the unimodal and fused estimates. (D–F) Schematized temporal relations between two stimuli. (D) When stimuli are presented with large temporal discrepancy, they are typically perceived as independent events and are processed separately. (E) When auditory–visual stimuli are presented with no or little temporal discrepancy, they are typically perceived as originating from the same source and their spatial evidence is integrated (fused). (C) When the temporal discrepancy is intermediate, causal inference can result in partial integration: the perceived timings of the two stimuli are pulled towards each other but do not converge.

Alt-text: Box 3

Evidence for which brain regions may reflect computational processes comes from a recent magnetoencephalography (MEG) study [87]. Streams of visual flickers and auditory tones were concurrently presented (at one of four different presentation rates), and participants categorised the rate of either the visual or auditory stream [87]. The study identified early cross-modal auditory-to-visual influences in primary visual cortex, which is in line with the earlier studies showing early cross-modal influences in primary sensory cortices [6,10]. In addition, by using representation similarity analysis, the authors observed a systematic progression from segregated unimodal representations (~100 ms visual and ~140 ms auditory), to fused multimodal representations (~180–260 ms), to causal inference (~620 ms). A recent EEG study investigating the temporal dynamics of Bayesian Causal Inference [86] provided a similar picture, although the precise timings of the effects were not consistent. Visual and auditory stimuli could appear within any number of simultaneously presented four frames, and participants were then prompted to report the number (1–4) of visual or auditory stimuli. Event-related potentials showed an early auditory–visual interaction effect starting at ~70 ms after stimulation onset. By combining Bayesian modelling with EEG representational similarity analysis, the authors also noted a progression from segregated unimodal representations (~100 ms), to fused multimodal representations (~200–300 ms), to causal inference (~400 ms). Moreover, the authors provided first evidence that prestimulus oscillatory alpha power as well as phase correlated with an observer’s prior belief about the causal structure of the world.

Thus, research based on both oscillatory activity and computational modelling suggest early cross-modal influences. Interestingly, however, although the computation work reveals early cross-modal influences, it also suggests a hierarchical view for integrating signals across modalities. Although the timings across studies do not match perfectly, they are consistent in suggesting a slow and protracted process of modality integration and causal inference. It will be worth investigating these timings in greater depth, to understand whether the results so far may reflect limitations in the sensitivity of analysis methods. It will also be interesting to understand whether there exist very early cross-modal influences, as suggested by oscillation research, that are independent of cross-modal mechanisms that are specifically related to integrating signals into multisensory representations.

## Future Directions

A growing body of evidence suggests that cross-modal influences in primary sensory cortices are mediated by the synchronisation of neural oscillations. This synchronisation may be driven by cross-modal phase reset, neural entrainment, or a combination of the two mechanisms. Both phase reset and neural entrainment are adaptive mechanisms and enable mutual influences and the flexible integration of multiple sensory stimuli across multiple time scales [15,63,90,91].

One important open question is whether and how the synchronisation of neural oscillations across sensory cortices is influenced by individual differences. These may arise from differences in people’s intrinsic brain rhythms as well as a result of their experience. Interestingly, when participants are asked to detect subtle gaps within a rhythmic auditory stimulus, performance shows that individuals differ from one another in their lag between stimulus-to-behavioural entrainment [38,49]. The next step would be to probe whether there are consistent individual differences in the timings of cross-modal entrainment, and whether they have functional consequences for cross-modal influences and integration. So far, one study has highlighted the importance of individual differences in cross-modal phase reset by demonstrating that the periodicity in phosphene perception was related to a participant’s individual alpha frequency [30]. Therefore, to understand how phase alignment facilitates cross-modal influences, future studies need to take account of individual differences. For external rhythmic stimulation, this could be done by presenting targets at multiple phase delays. One could then determine a participant’s preferred phase lag a priori and investigate whether cross-modal influences are enhanced when targets are presented in phase with a participant’s preferred phase delay and impeded when presented out of phase.

In addition to individual differences related to intrinsic factors, experience may also shape the temporal parameters of cross-modal influences. For example, long-term musical training was shown to increase the temporal sensitivity for auditory–visual synchrony [92]. Functional imaging revealed the effect to be accompanied by modulation in multisensory cortex (STS) and cerebellar and premotor regions. Electrophysiological studies will prove informative in testing for changes in the strength and precision of synchronisation of cross-modal signals related to long-term experience. Effects on cross-modal influences can also develop quickly. In temporal recalibration experiments, participants’ perception of the timing between events changes as they adapt to regular intervals (e.g., [93,94]). Studying the modulation of synchronisation in recalibration studies will reveal interesting insights. For instance, one EEG study found phase alignment of oscillatory activity in tandem with auditory–visual lag adaptation [93].

The synchronisation of neural oscillations likely facilitates information transfer across sensory cortices by linking information in local and large-scale brain networks [2,15,63,90,91]. As noted earlier, at least three distinct types of anatomical pathways may support cross-modal influences: indirect input from higher order multimodal cortical areas (e.g., IPS, STS, and PFC); connections through multimodal subcortical regions (e.g., superior colliculus and the pulvinar nucleus of the thalamus) [10., 11., 12.]; and possibly direct lateral connections between unimodal cortices [13]. Depending on the nature of the stimulation and/or the tasks, different pathways may come into play and, in some cases, interact. One interesting line of future research will be to understand the different oscillatory signatures of the various pathways and to investigate the extent to which synchronisation facilitates cross-modal influences and sensory integration within and between them.

A prevailing view, as presented in this review, is that selection and routing of sensory information and ultimately cross-modal integration, is facilitated by the synchronisation of sustained rhythmic fluctuations. However, recent studies have highlighted the possibility that, in some cases, information is instead coded through **transient burst-events** [95,96]. While burst-events might enable transient cross-modal communication through brief synchronisation across neuronal ensembles, it is less clear how they might enable longer-lasting information transfer. Therefore, it would be fruitful to test whether individual stimuli may synchronise brain activity through phase reset mechanisms triggered by burst events, whereas longer lasting rhythmic stimulation may synchronise brain activity through the entrainment of more sustained oscillations. To identify and accurately separate burst events and continuous neural oscillations, and to test their relative contributions to cross-modal influences, we need analysis tools with better temporal resolution so that we can characterise and quantify the real-time rhythmic structure of brain activity. Typical Fourier-based methods rely on temporal windowing and/or on imposing temporally extended filters in the form of wavelets. Happily, new analytical approaches are being applied to brain signals that can characterise moment-to-moment fluctuations in oscillatory activity. The Empirical Mode Decomposition (EMD) uses a data-driven approach to separate time series into their various constituent frequency modes [97], and is beginning to be applied to analyse how instantaneous phase and morphology of activity in different frequency bands influence neural activity and behaviour [98]. Hidden Markov modelling (HMM) is also being used to increase substantially the temporal resolution of M/EEG analysis [99]. HMM-based analyses can flexibly incorporate parameters, such as phase of different oscillations, to describe and compare different brain states [99,100] and evaluate their functional consequences. Such methods, combined with increased spatial resolution, for instance as obtained with ECoG and depth-electrode recordings, should significantly enhance our ability to test the involvement of short-lived neural bursts and more prolonged oscillations in phase-reset and neural entrainment mechanisms in selecting, routing, and integrating information across different neural circuits to guide adaptive performance.

Presently, most studies investigating cross-modal influences in humans focus on the interactions between the auditory and visual modalities. The temporal order in which auditory and visual stimuli occur has an important role in how we perceive them (for a review, see [21,101]). Looking ahead, a more detailed picture of cross-modal influences can be achieved by incorporating the somatosensory, as well as other sensory modalities.

Studies highlighted in this review have investigated neurotypical populations. However, we have also learned that individual differences, due to intrinsic physiological differences or to experience, can impact the parameters of cross-modal influences. Therefore, a fuller understanding of cross-modal influences may emerge by comparing effects across the lifespan or in populations with sensory deficits, such as patients with visual or hearing impairments. To date, there is no consistent picture regarding putative systematic changes in mechanisms of cross-modal influences as our senses mature in early life or degenerate in later years. Regarding clinical populations, early EEG studies testing patients with cochlear implants have suggested that cross-modal functional activation patterns, such as visual takeover, are maladaptive for later sensory restitution [102]. However, more recent work clearly shows that cross-modal cortical reorganisation can be beneficial [103., 104., 105.]. A lifespan developmental approach will provide a strong test of the functional contributions of oscillatory synchronisation in support of multisensory processing.

## Concluding Remarks

There is ample evidence that cortical cross-modal influences start early, at the level of primary sensory cortices, inviting us to re-examine how we conceptualise unimodal cortices. Computational modelling is likely to have an important role in helping to understand whether and how different types of oscillatory processes support cross-modal influences and integration. Regulation of the synchronisation of rhythmic, oscillatory activity in the brain has been proposed to facilitate the selection, routing, and integration of neural activity within sensory modalities [19,20]. Here, we have considered evidence suggesting that synchronisation of neural oscillations also has a vital role in facilitating the transfer and integration of sensory information across modalities. We focused on two oscillation-related mechanisms that can promote cross-modal synchronisation: phase reset and neural entrainment. We have suggested that these mechanisms for cross-modal synchronisation display flexibility and are modulated by task goals and temporal expectations. In particular, evidence thus far suggests that low-frequency neural oscillations in the delta, theta, and lower alpha range provide permissive temporal windows for cross-modal influences [10,25,34,68].

Several important and exciting questions remain open for further research (see Outstanding Questions). Our success in revealing the fundamental principles of cross-modal influences will depend on considering interindividual differences in our experimental tasks, comparing oscillatory mechanisms across development and in clinical populations, and developing new analysis methods to individuate and characterise the duration and morphology of rhythmic brain activity in different frequency bands.

Outstanding Questions How do individual differences, such as experiences or intrinsic brain rhythms, modulate cross-modal influences in general and phase alignment of neural oscillation in particular? For instance, do individual differences, such as musical experience, influence the quality of cross-modal phase alignment?

How is information transferred across sensory cortices? Is cross-modal information routed through sustained rhythmic fluctuations or via transient burst events (or both)? New methodological approaches will help to address this question.

Most studies have focused on probing cross-modal influences with auditory or visual stimulation. Does (rhythmic) somatosensory stimulation prove equally effective to influence perception in the visual and/or the auditory modalities?

What is the modulatory role of top-down factors, such as goals, expectations, or memory, on cross-modal influences? What are the oscillatory signatures underlying such cognitive top-down processes and which cortical networks are involved?

How do cross-modal influences between sensory cortices develop over the lifespan?

What are the oscillatory signatures of cross-modal processing in clinical populations with sensory deficits, such as individuals with hearing or visual impairments?

How can we bridge the gap between computational modelling work and findings based on oscillation-related mechanisms, such as phase reset and neural entrainment?

Alt-text: Outstanding Questions

## Glossary

**Bayesian Causal Inference**: describes how an observer should arbitrate between information integration and segregation to compute an estimate of the sources of incoming sensory signals. It explicitly models the potential causal structures (i.e., common or independent sources) that could have generated input from different sensory modalities.
**Cross-modal influence**: how processing of a modality-specific sensory stimulus, that is a stimulus presented to one sensory modality (e.g., sound), affects neural and behavioural processing associated with another sensory modality (e.g., vision).
**Cross-modal phase reset**: refers to the process by which the phase of ongoing neural oscillations in one sensory modality can be ‘reset’ by a transient event in another sensory modality.
**Dynamic-attending theory (DAT)**: proposes that attention dynamically waxes and wanes according to rhythmic stimulation to optimise sensory processing of events predicted in time.
**Neural entrainment**: the process through which two or more self-sustained oscillators become coupled (see Box 2 in the main text).
**Neural oscillations**: reflect rhythmic fluctuations between high and low excitability states in neuronal circuits. Neural oscillations are typically measured according to a hierarchy of distinguishable frequency bands, including delta (1–4 Hz), theta (4–8 Hz), alpha (8–12 Hz), beta (10–30 Hz), and gamma (30–100 Hz). Neural oscillations can be quantified in terms of frequency, amplitude, and phase.
**Phase**: indicates a particular point in an oscillatory cycle between 0 and 2 pi, corresponding to trough, rising slope, peak, and so forth.
**Temporal expectation**: the state of the neural or cognitive system associated with the predicted timing of an event.
**Top-down process**: modulation of ongoing stimulus analysis by signals carrying information about the internal state of the individual (e.g., task goal or expectation).
**Transient burst-events**: a brief boost of high power that arises transiently and intermittently. Transient burst events can not only be described by their frequency and amplitude, but also by their rate, timing, duration, and shape.
